# Can’t Understand It

**Published:** 1886-08

**Authors:** 


Can't Understand It.—There are many things we cannot understand. One is, how a person can reconcile to his conscience the reading of a journal month after month without remitting the amount of subscription, or notifying the editor of his wish to discontinue accompanied by arrearages to date. To such of our readers we would say, if you wish the Journal continued please remit the subscription price; if you wish it discontinued please inform us of the fact on a postal card.—[Exchange.

